# The Diagnostic Value of Whole-Exome Sequencing in a Spectrum of Rare Neurological Disorders Associated with Cerebellar Atrophy

**DOI:** 10.1007/s12035-023-03866-y

**Published:** 2023-12-28

**Authors:** Engy A. Ashaat, Hoda A. Ahmed, Nesma M. Elaraby, Alaaeldin Fayez, Ammal M. Metwally, Mona K. Mekkawy, Dalia Farouk Hussen, Neveen A. Ashaat, Rasha M. Elhossini, Heba Ahmed ElAwady, Randa H. A. Abdelgawad, Mona El Gammal, Mohamed Ahmed Al Kersh, Dina Amin Saleh

**Affiliations:** 1 Clinical Genetics Department, Human Genetics and Genome Research Institute, National Research Centre, Cairo, Egypt; 2 Medical Molecular Genetics Department, Human Genetics and Genome Research Institute, National Research Centre, Cairo, Egypt; 3 Molecular Genetics and Enzymology Department, Human Genetics and Genome Research Institute, National Research Centre, Cairo, Egypt; 4 Community Medicine Research Department, Medical Research and Clinical Studies Institute, National Research Centre, Cairo, Egypt; 5 Human Cytogenetic Department, Human Genetics and Genome Research Institute, National Research Centre, Cairo, Egypt; 6 Human Genetics, Ain Shams University, Cairo, Egypt; 7 Pediatrics Department, Fayoum University Hospitals, Faiyum, Egypt; 8 Ophthalmology Department, Ain Shams University, Cairo, Egypt; 9 Orthopedic Department, Ain Shams University, Cairo, Egypt; 10 Pediatric Department, Faculty of Medicine, Ain Shams University, Cairo, Egypt

**Keywords:** Neurodegenerative disorders, Neurological disorders, Novel variants, Cerebellar atrophy, WES

## Abstract

Several neurological disorders, neurodevelopmental disorders, and neurodegenerative disorders have a genetic element with various clinical presentations ranging from mild to severe presentation. Neurological disorders are rare multifactorial disorders characterized by dysfunction and degeneration of synapses, neurons, and glial cells which are essential for movement, coordination, muscle strength, sensation, and cognition. The cerebellum might be involved at any time, either during development and maturation or later in life. Herein, we describe a spectrum of NDDs and NDs in seven patients from six Egyptian families. The core clinical and radiological features of our patients included dysmorphic features, neurodevelopmental delay or regression, gait abnormalities, skeletal deformities, visual impairment, seizures, and cerebellar atrophy. Previously unreported clinical phenotypic findings were recorded. Whole-exome sequencing (WES) was performed followed by an in silico analysis of the detected genetic variants’ effect on the protein structure. Three novel variants were identified in three genes *MFSD8*, *AGTPBP1*, and *APTX*, and other previously reported three variants have been detected in “*TPP1*, *AGTPBP1*, and *PCDHGC4*” genes. In this cohort, we described the detailed unique phenotypic characteristics given the identified genetic profile in patients with neurological “neurodevelopmental disorders and neurodegenerative disorders” disorders associated with cerebellar atrophy, hence expanding the mutational spectrum of such disorders.

**Supplementary Information:**

The online version contains supplementary material available at 10.1007/s12035-023-03866-y.

## Introduction

Inherited complex genetic disorders are common in Egypt and constitute a public health burden [1]. Neurodevelopmental disorders affect more than 3% of the pediatric population in many of their severe chronic and progressive forms that are expected to have an underlying genetic etiology that remains undiagnosed despite all available genetic tools [2].

Neurological disorders such as neurodevelopmental disorders and neurodegenerative disorders (NDs) are characterized by extreme genetic heterogeneity [3, 4] implying that whole-exome sequencing (WES) is the most appropriate first-tier test to cover more analyzed genes in neurogenetic disorders [5].

Neurodegenerative disorders are a heterogeneous group of mostly genetically determined diseases that result in progressive loss of neuronal structures or functions in different areas of the central and peripheral nervous system with a resultant loss of the previously acquired motor, sensory, and cognitive functions [4]. In the pediatric age group, the accretion of new developmental milestones does not exclude the existence of an ND disorder. On the clinical level, neurodegenerative disorders share similar manifestations including visual and hearing impairment, seizures, skeletal deformities, feeding, and intellectual difficulties [6]. Therefore, reaching a specific diagnosis could be quite challenging in the pediatric age group especially in resource-limited countries due to several reasons such as the ability of the clinicians to discriminate between the loss of a previously acquired and a delay in the achievement of specific developmental milestones, lack of expertise, and the long list of unaffordable potential investigations including molecular genetic analysis [7]. On the radiological level, the posterior fossa structures show variable degrees of involvement suggesting the period of affection such as growth cessation “prenatal,” growth cessation with concurrent atrophy “prenatal and postnatal,” or either stationary or progressive cerebellar atrophy “postnatal” [8]. On the molecular and biochemical level, NDs are characterized by depositions of misfolded, toxic conformations of various proteins, which generally accumulate to form insoluble deposits [9].

In this study, we will review the clinical features and radiological findings to explore the molecular and mutation spectrum in seven Egyptian patients with neurological “neurodevelopmental disorders and neurodegenerative disorders” disorders with an overlapping phenotype. We employed WES to screen the mutations and investigate the genotypic and phenotypic heterogeneities of molecularly characterized patients. With this, we aim to provide a better understanding of neurological disorders with an underlying genetic etiology among clinicians especially in resource-limited countries to help them offer appropriate management, prognosis expectations, and proper genetic counseling.

## Material and Methods

### Ethical Approval

The ethical approval was granted by the Medical Research Ethics Committee of the National Research Centre (NRC), Cairo, Egypt (Number: 932702021) according to the Declaration of Helsinki. Informed consent was obtained from the patient’s parents.

### Patient Enrollment and Clinical Analysis

Seven patients (five males and two females) were recruited from the Multiple Congenital Anomalies Clinic (MCAs), Clinical Genetics Department, National Research Centre (NRC). Patients either presented primarily at the MCAs or have been referred by participating pediatric neurologists or ophthalmologists for further evaluation and workup completion.

Patients presented with developmental delay, neurodevelopmental regression (NDR), neurobehavioral disorders, visual or hearing impairment, short stature, abnormal gait, skeletal abnormalities, seizures, or family history of early unexplained death with uneventful perinatal or postnatal course were included in this study.

Once patients were identified as having possible neurological neurodevelopmental disorders or neurodegenerative disorders disorder, the caregivers were counseled about the possible genetic diagnosis and the required genetic test. The initial evaluation of patients comprised detailed history taking including “family history of a similar condition or other genetic disorders and perinatal history of possible prenatal insult or postnatal complications”, neurodevelopmental milestones assessment, and thorough physical and neurological examination. Patients with a perinatal history of maternal infection or postnatal complications such as kernicterus, meningitis, stroke, posterior fossa surgery, radiotherapy, head trauma, or suspected primary mitochondrial disorders were excluded from the study.

**Fig. 1:**
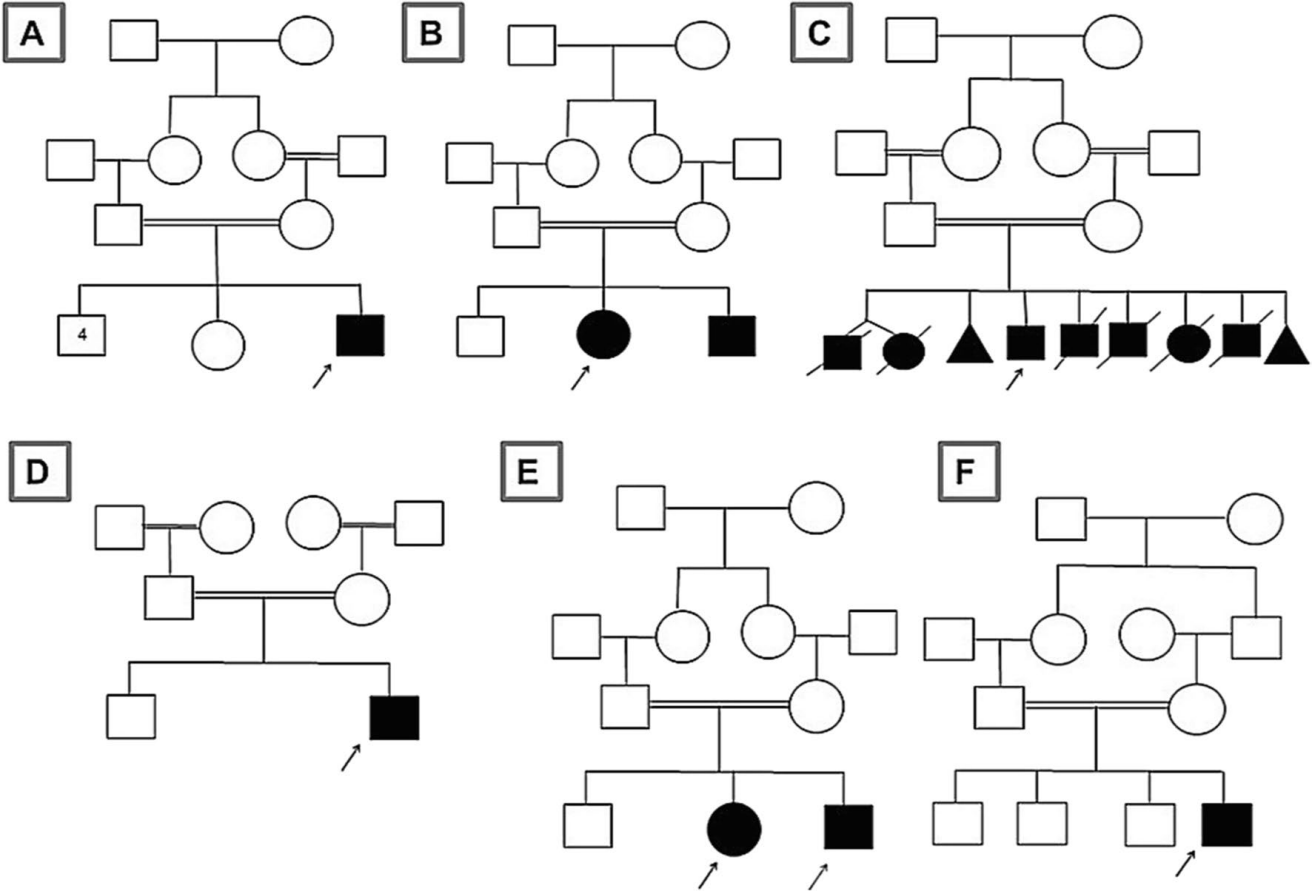
Family pedigrees of the six studied unrelated families. **A** P1, **B** P2, **C** P3, **D** P4, **E** P5&P6, **F** P7

Detailed demographic data revealed positive consanguinity of all the examined patients and positive family history in four patients. The age range was from 1.5 to 18 years old at presentations. Anthropometric measurements including “head circumference, weight, and height/length” were plotted according to the recommendation of the International Biological Program (IBP) and showed underweight in two patients while height was not evaluated in one patient due to the associated scoliosis and joint contractures [10]. The detailed family pedigree is shown in Fig. 1. Detailed patients’ demographic data and anthropometric measurements are shown in Table 1. A special emphasis on the presence of dysmorphic features, skeletal deformities, and a review of other body systems was carried out to analyze the phenotypic presentation of the index patients. Physical and neurological examination was carried out for both parents and available siblings. The core clinical presentation of our patients included dysmorphic features (two patients), neurodevelopmental delay or regression (seven patients), gait abnormalities (three patients), skeletal deformities (five patients), visual impairment (seven patients), and seizures (five patients). Additional clinical features and diagnostic workup are described in Table 2.

**Table 1:** Patient’s demographic data, anthropometric measurements and similarly affected siblings

Patient	Gender	Age at onset (years)	Current age (years)	Parental consanguinity	Anthropometric measurements	Other affected family members
P1	Male	3	6	1st cousins	- OFC: appropriate - Wt: appropriate - Ht/Lt: appropriate	None
P2	Female	1	1.5	1st cousins	- OFC: microcephaly - Wt: underweight - Ht/Lt: appropriate	1 male & 1 female siblings
P3	Male	2	18	1st cousins	- OFC: appropriate - Wt: underweight - Ht/Lt: N/A (severe contracture deformities and scoliosis)	5 male & 2 female siblings
P4	Male	2	9	1st cousins	- OFC: appropriate - Wt: appropriate - Ht/Lt: appropriate	None
P5	Male	6	12	1st cousins	- OFC: appropriate - Wt: appropriate - Ht/Lt: appropriate	1 female sibling
P6	Female	7	13	1st cousins	-OFC: appropriate -Wt: appropriate -Ht/Lt: appropriate	1 male sibling
P7	Male	1.5	5	2nd cousins	- OFC: appropriate - Wt: appropriate - Ht/Lt: appropriate	None

*OFC* occipito-frontal circumference, *Wt* weight, *Ht* height, *Lt* length

**Table 2:** Patient’s clinical features and diagnostic work up

Patient	Diagnosis	Symptoms	Dysmorphic features	Neurological examination	Eye manifestation	Skeletal deformity	VEP/ERG)	ECHO	EEG	Brain MRI	Nucleotide Change Protein Change	ACMG Classification
P1	NCL type 2	- GDD - Progressive visual loss - Seizures	No	- Spastic quadriplegia - Brisk deep tendon reflexes	Poor eye contact and visual tracking	Scoliosis	Bilateral poor retinal function	Normal	Abnormal	Cerebral & cerebellar atrophy Hypoplastic corpus callosum	C.1145 + 2 T > G p.? TPP I(CLN2)	Pathogenic
P2	NEDGS	- GDD - Visual decline - Seizures - Dyspnoea on exertion	- Synophrys Short philtrum, thick lips, micrognathia, low set ears)	Hypertonia/brisk reflexes	Oculomotor apraxia	Scoliosis Bilateral hip dislocation	NI	ASD	Normal	Cerebellar vermis hypoplasia	c.1463C > T p. Ala488Val PCDHGC4	Likely Pathogenic
P3	CONDCA	- GDD - Seizures -Intellectual difficulties - Skeletal deformities	- Course facial features (bulbous broad nose, thick lips, large ears) - Narrow forehead - Thick eyebrows	- Spasticity - Brisk deep tendon reflexes - Contractures	- Poor eye contact - Hypometric saccades - Oculomotor apraxia	Scoliosis Contracture deformities	Bilateral poor retinal function	Normal	Abnormal	Cerebellar atrophy	c.2650A > C* P. Thr884Pro AGTPBP1	Likely Pathogenic
P4	AOA1	- Progressive NDR - Intellectual disability - Jerky hand movements - Abnormal gait - Dyspnea on exertion	No	- Progressive ataxia - Intention tremors - Spasticity of lower extremities - Brisk deep tendon reflexes	-Hypometric saccades -Bilateral myopic astigmatism Oculomotor apraxia	No	Bilateral optic nerve dysfunction	CHD (Aortic and tricuspid valve regurgitation)	Not done	Cerebral & cerebellar atrophy Hypoplastic corpus callosum	c.635C > T* P. Ala212Val APTX	VUS
P5	NCL type 7	-Intellectual difficulties - NDR -Photosensitive epilepsy - Abnormal gait - Visual decline	No	- Poor fine motor skills - Clumsy gait - Poor coordination (finger to nose test)	Poor vision	No	Retinal dystrophy (rod/cone dystrophy) Optic nerve dysfunction	NI	Abnormal	Cerebellar atrophy	c.638C > A* p. Pro213Gln MFSD8	Pathogenic
P6	NCL type 7	- Intellectual disability - NDR Photosensitive epilepsy - Visual decline -Abnormal gait and posture	No	- Poor fine motor skills - Clumsy gait - Poor coordination (finger to nose test)	Poor vision	Scoliosis	Retinal dystrophy Optic nerve dysfunction	NI	Abnormal	Cerebellar atrophy	c.638C > A* p. Pro213Gln MFSD8	Pathogenic
P7	CONDCA	- Intellectual disability - GDD - Feeding difficulties	No	- Wheel chair bound (GMFCS level IV Truncal hypotonia - Weak hand grip and poor fine motor skills Weakness and spastic lower limbs Wasting of leg muscle - Areflexia in lower extremities	- Strabismus (wearing eye glasses) - Oculomotor apraxia - Eye lid twitching	Knee and Achilles tendon contractures - Bilateral tightness of the Achilles tendon	Bilateral optic nerve dysfunction ERG N/A	Normal	NI	Cerebellar atrophy	c.1534A > G p. Thr512Ala *AGTPBP1*	VUS

*AOA1* ataxia-ocular apraxia type 1, *CONDCA* childhood-onset neurodegeneration with cerebellar atrophy, *NCL* neuronal ceroid lipofuscinosis type 2, *NEDGS* neurodevelopmental disorder with poor growth and skeletal anomalies, *GDD* global developmental delay, *NDR* neurodevelopmental regression, *CHD* congenital heart disease, *ASD* atrial septal defect, *NI* not indicated, *ACMG* American College of Medical Genetics and Genomics, *VUS* variant of unknown significance

^*^Novel variant

### Other Ancillary Tests

A skeletal survey including plain X-rays of the “skull, spine, pelvis, short and long bones,” brain magnetic resonance imaging (MRI), electroencephalogram (EEG), electroretinogram (ERG), visual evoked potential (VEP), and echocardiography (Echo) was carried out whenever indicated.

### Cytogenetic Analysis

Karyotype analysis was performed for all patients to exclude the presence of any chromosomal abnormalities. Chromosomal preparations were done from peripheral blood samples collected on lithium heparin vacutainers, following standard protocols [11].

### Molecular Analyses

#### DNA Extraction and WES

A total of 3-ml venous blood was collected in EDTA tubes from all patients and their available family members. Genomic DNA was extracted from peripheral blood samples of all participants using the QIAamp DNA Mini Kit (Qiagen, Hilden, Germany). The quality and quantity of DNA samples of patients were assessed using fluorometric Denovix Qubit™ dsDNA BR Assay Kit (ThermoFisher, Waltham, MA, USA). DNA samples were sequenced by using the Twist Human Core Exome Plus kit (Twist Bioscience, San Francisco, CA, USA) and Illumina NovaSeq 6000 system (Illumina, San Diego, CA, USA) according to the manufacturer’s protocol. Libraries were prepared in paired-end mode (2 × 100 bp) for an output of 6 GB per sample, and an average coverage of 50 × . Sequencing reads were demultiplexed using Illumina Genes 2022, 13, 1056 4 of 24 bcl2fastq (2.20), and adapter sequences were trimmed using Skewer (version 0.2.2) [12]*.* The quality of the generated FASTQ files was analyzed with FastQC software (version 0.11.5; Illumina, San Diego, CA, USA). Variant Annotation and Filtration PhenoDB tool were used to annotate Vcf files using ANNOVAR [13]*.* Variants were filtered based on the depth of coverage and minor allele frequencies (MAF) (less than 1% MAF) in large population databases, including dbSNP [14], 1000 Genomes Project [15], and the Genome Aggregation Database (gnomAD v2.1.1) [16].

#### Variant Segregation

Sanger sequencing was used to confirm that prioritized variants segregated consistently among parents and available family members according to the predicted mode of inheritance. We designed primers targeting exons that harbor the filtered variants of interest using the Primer3 tool [17]. PCR was carried out as previously described. Reactions were sequenced according to the manufacturer’s recommendation using the Big Dye Termination kit (Applied Biosystems, Waltham, MA, USA) and ABI Prism 3500 Genetic Analyzer (Applied Biosystems, Waltham, MA, USA). Variants were named based on Human Genome Variation Society nomenclature recommendations [18]. The standards of the American College of Medical Genetics and Genomics (ACMG) were used to classify the level of variant pathogenicity, i.e., pathogenic, likely pathogenic, variant of unknown significance (VUS), benign, or likely benign [19].

#### Multi-scale Computational Analysis

The possible biological effect of all missense variants was done using multi-scale computational analysis tools considering all probable pathogenicity relevant aspects. Multi-scale computational analysis approach supports multiple entry variants for annotation and analysis permitting the closest true pathogenicity implication to be concluded. To explore the functional network among all variants harboring genes in this study, the functional enrichment and protein-protein enrichment analyses were carried out using GeneMania and STRING servers.

All the variants were described using chromosomal reference sequences according to HGVS recommendations and were checked by LUMC mutalyzer v. 3.0.4 according to GRCH38 human genome assembly. All mentioned genes were described according to HGNC nomenclature.

## Results

### Clinical Features and Phenotyping

Table 1 displays the patient’s anthropometric measurements, demographic information, and siblings who are also affected.

**Fig. 2:**
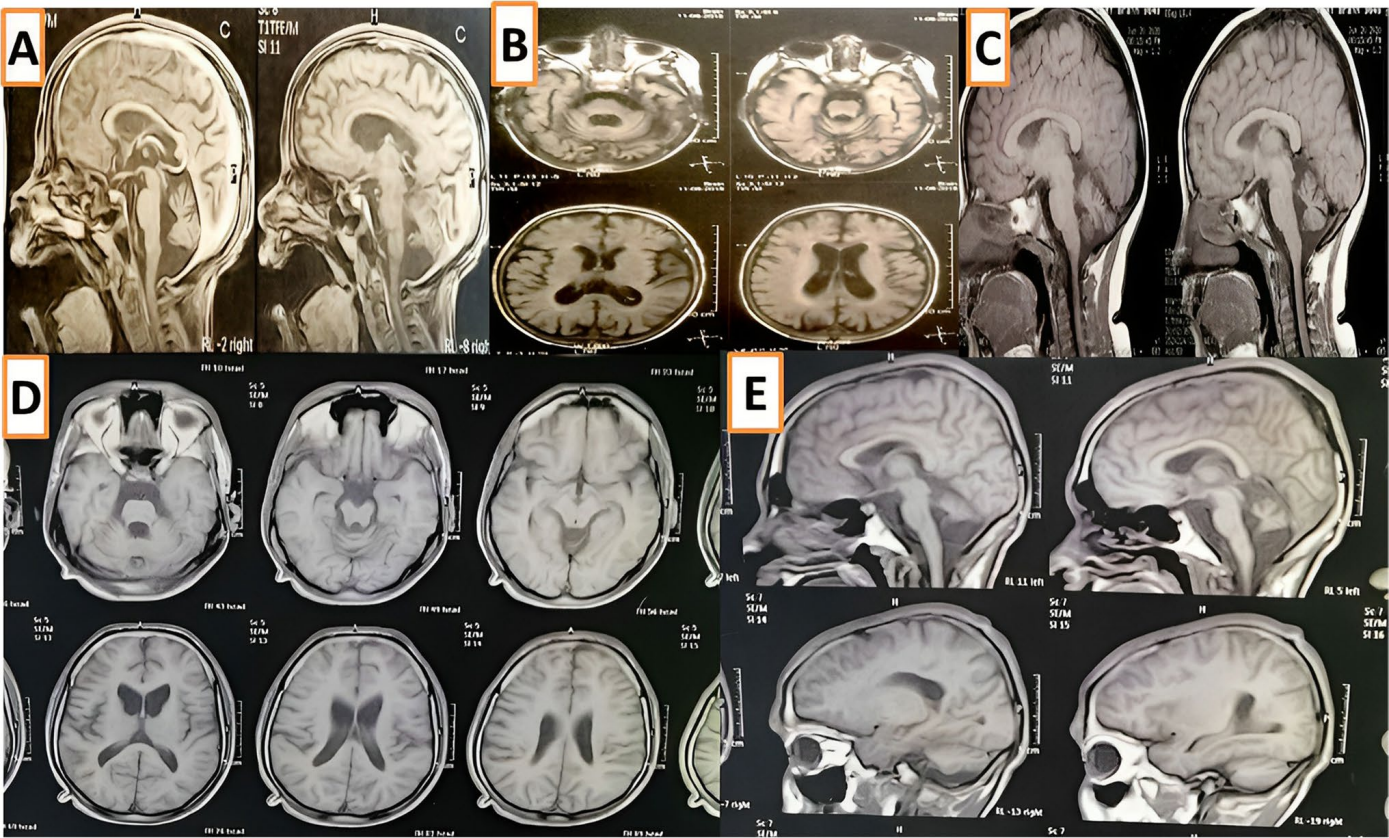
Brain MRI findings of the studied patients. P1 (**A**, **B**) cerebral and cerebellar atrophy and hypoplastic corpus callosum; P2 (**C**, **D**) cerebellar vermis hypoplasia; P3 (**E**) cerebellar atrophy

Patient 1 presented with global developmental delay (GDD), language impairment, seizures, progressive loss of vision with poor ocular fixation, progressive spastic quadriplegia, and scoliosis. A maternal history of recurrent spontaneous abortion was recorded. The ancillary tests-ERG showed retinal dysfunction, EEG showed interictal generalized epileptiform activity, and brain MRI showed cerebral and cerebellar atrophy and hypoplastic corpus callosum (Fig. 2A, B).

Patient 2 presented with dysmorphic facial features including “synophrys, short philtrum, thick lips, micrognathia and low set ears,” poor ocular fixation and oculomotor apraxia, delayed motor development, spasticity, generalized tonic-clonic seizures, atrial septal defect (ASD), scoliosis, and bilateral hip dislocation. The family history of a similarly affected sister was reported. The ancillary tests-EEG was normal, and brain MRI showed cerebellar vermis hypoplasia (Fig. 2C, D).

**Fig. 3:**
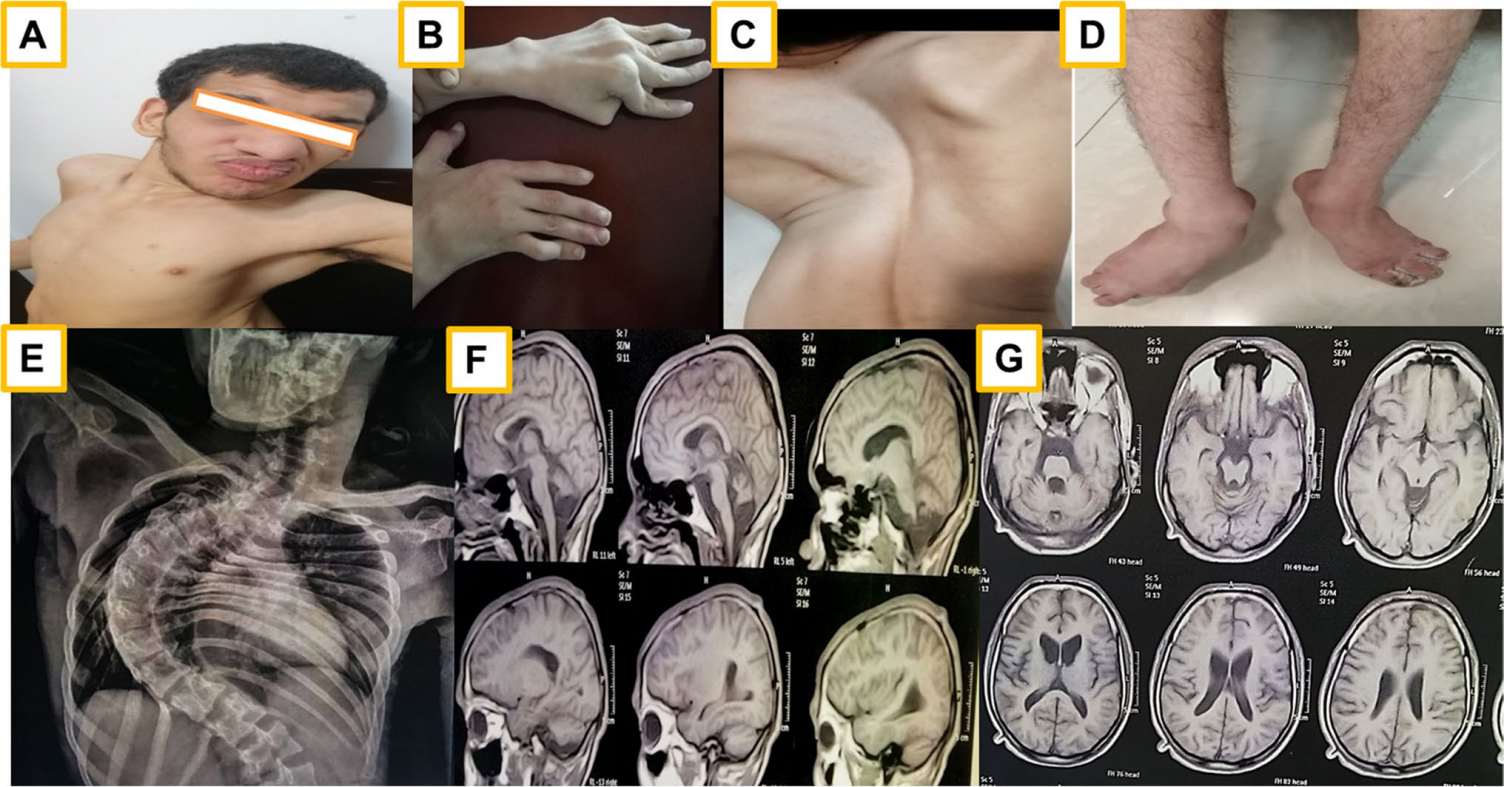
Patient 3—phenotypic dysmorphic features, scoliosis, skeletal deformities, and fungal feet infection. X-ray chest PA view showing severe scoliosis and ribs crowding (**E**), MRI brain (**F**, **G**) showed cerebellar atrophy

Patient 3 presented with GDD, dysmorphic facial features “course facies, narrow forehead, thick eyebrows, broad bulbous nose, short philtrum, thick lips, and large ears,” oculomotor apraxia, and seizures. He had multiple skeletal deformities “arachnodactyly, bilateral hyperextensibility of the interphalangeal joints, bilateral low inserted thumb, toes camptodactyly, bilateral prominent heel, severe scoliosis and joint contractures” and bilateral fungal infection of both feet (Fig. 3). The ancillary tests-ERG showed retinal dysfunction, EEG was abnormal, and brain MRI showed cerebellar atrophy (Fig. 2E).

Patient 4 presented with intellectual disability, progressive ataxia “started at the age of 5 years,” intension tremors, and oculomotor apraxia. He had also aortic regurgitation with a thickened valve. The ancillary tests-ERG showed retinal dysfunction, VEP showed bilateral optic nerve dysfunction, and brain MRI showed cerebral and cerebellar atrophy and hypoplastic corpus callosum (Fig. 2G, H).

Patients 5 and 6 (siblings) both presented with progressive NDR, intellectual disability, and seizures (photosensitive epilepsy). They were also noted to have progressive visual decline, poor visual hand-motor coordination, and abnormal clumsy gait. Patient 5 had focal to bilateral seizures with loss of awareness that were preceded by seeing different colors. His EEG showed a single burst of sharply contoured sharp waves over the right parasagittal area. Patient 6 had scoliosis and seizures “absence and generalized tonic and clonic” that were triggered by intense light. Her EEG showed generalized, fragmented, and focal rhythmic epileptiform discharges arising mainly from the right hemisphere. Their seizures were initially well controlled on levetiracetam, but later on, they showed a refractory course. Both patients had abnormal ERG/VEP as shown in Table 2. Both patients had cerebellar atrophy as shown in brain MRI findings in patient 5 (Fig. 2I, J).

Patient 7 presented with GDD, speech difficulties, and impaired cognition. He had feeding difficulties, strabismus, and oculomotor apraxia. He had a left-hand preference and poor fine motor skills. He had never been able to walk independently and exhibited bilateral knee contractures and bilateral tightness of the Achilles tendon. The ancillary tests-ERG/VEP showed bilateral optic nerve dysfunction, and brain MRI showed cerebellar atrophy (Fig. 2K).

### The Genetic Spectrum of NDs Patients

Exome analyses of the seven studied patients descending from six unrelated Egyptian families identified six different disease-causing variants in five genes; TPP1 (*NM_000391.4*), *MFSD8* (NM_001371596.2), *AGTPBP1* (NM_001330701.2), *APTX* (NM_001195248.2), and *PCDHGC4* (NM_018928.3) genes as displayed in Tables 2 and 3. All of these variants were missense except one defined as a splicing variant and according to ACMG classification criteria were classified as pathogenic variants. As well, the analysis confirmed that among these, three variants were not found in dbSNP, TGP, gnomAD exome, and ExAC databases or in our in-house database of 55 Egyptian exomes.

**Fig. 4:**
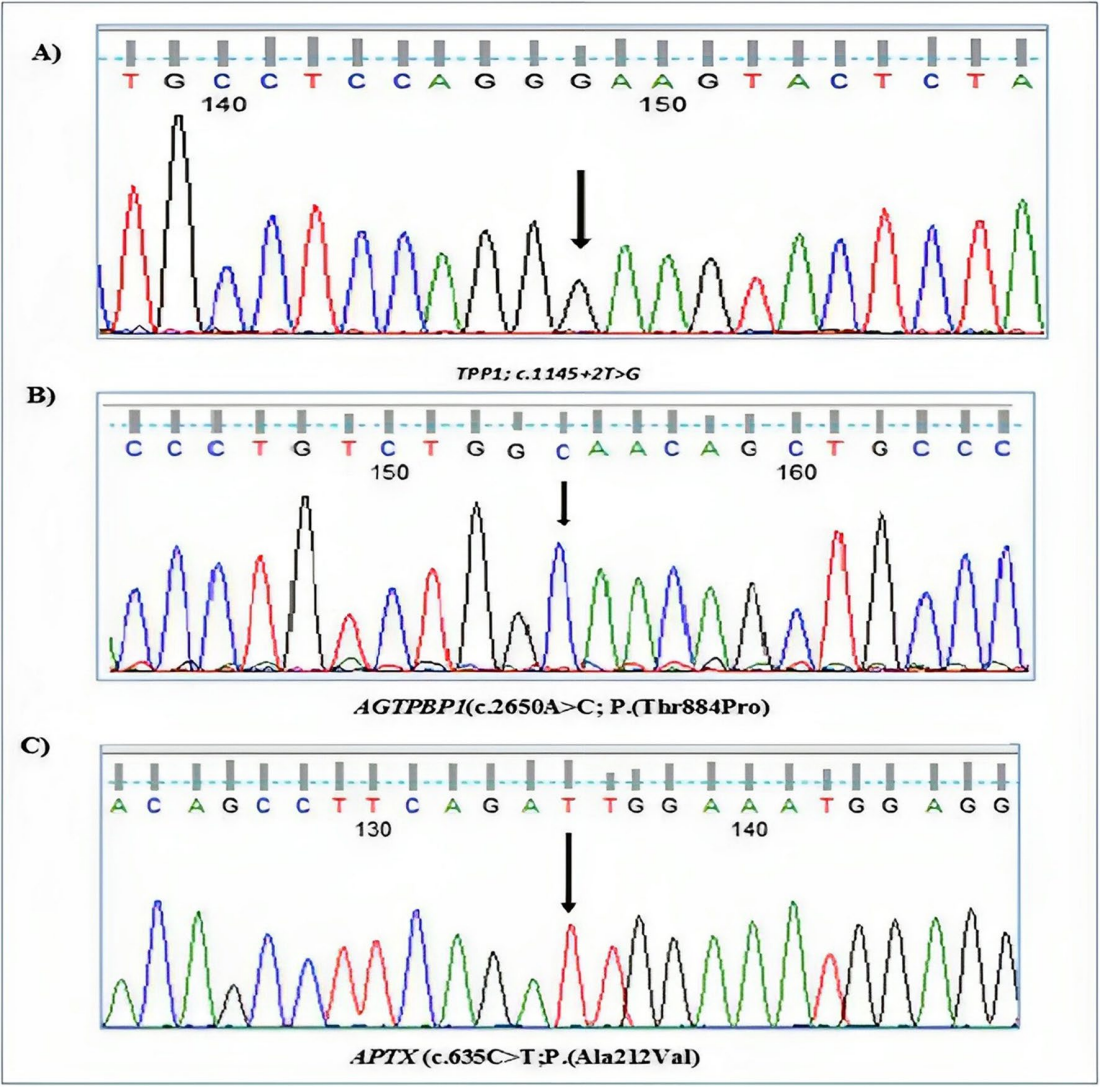
Sanger sequencing chromatograms of three patients (P1, P3, P4)

Sanger sequencing was performed to confirm that prioritized variants segregated consistently among parents and available family members according to the predicted mode of inheritance. The chromatograph for available patients who completed the follow-up was described in Fig. 4.

Patient 1 had a homozygous splicing variant in *TPP1* gene (C.1145 + 2 T > G). This variant is predicted to disrupt the highly conserved donor splice site of exon 9. Together with the clinical information and biochemical results, it is classified as pathogenic (class 1) according to the ACMG. The result is consistent with the genetic diagnosis of AR NCL type 2 (Fig. 4).

Patient 2 had a homozygous missense variant in the *PCDHGC4* gene (c.1463C > T; p.(Ala488Val). It is a likely pathogenic variant according to ACMG. Defects in *PCDHGC4* have been associated with NEDGS.

Patient 3 had a novel homozygous missense variant in the *AGTPBP1* gene (c.2650A > C; p.(Thr884Pro) substituting threonine residue for proline at position 884. It is a likely pathogenic variant according to ACMG. Pathogenic variants in the *AGTPBP1* gene are associated with AR CONDCA (Fig. 4).

Patient 4 had a novel homozygous missense variant in the *APTX* gene (c.635C > T; p.(Ala212Val) in exon 6 substituting alanine for valine at position 212. The pathogenic variant has previously been described as disease-causing AOA1. It is classified as a variant of uncertain significance (class 3) according to ACMG (Fig. 4).

Patients 5 and 6 are affected siblings, both of whom had a novel homozygous missense variant in *MFSD8* gene (c.638C > A; p.(Pro213Gln) substituting proline residue for glutamine at position 213. The homozygous state of this variant has been confirmed by parental targeted testing. It is classified as a variant of uncertain significance (class 3) according to ACMG. Pathogenic variants in *the MFSD8* gene are associated with AR NCL type 7.

Patient 7 had a homozygous missense variant in the *AGTPBP1* gene (c.1534A > G; p. (Thr512Ala) causing an amino acid change from Thr to Ala at position 512. It is classified as a variant of uncertain significance (class 3) according to the recommendations of Centogene and ACMG. Pathogenic variants in the *AGTPBP1* gene are associated with AR CONDCA.

### Computational Analysis Implications

All the used computational tools and the corresponding implications are shown in Supplementary Table 1. The functional enrichment analysis is shown in (Supplementary Fig. 1).

## Discussion

In this study, we described the detailed phenotypic, radiological, and molecular findings of seven Egyptian patients presenting with neurological Neurodevelopmental disorders or neurodegenerative disorders disorder. All parents were first-degree cousins, highlighting the impact of consanguineous marriage on the increased rate of AR genetic disorders reported in our country [20].

Genetic diagnostic testing based on exome sequencing revealed three novel variants in “*MFSD8* (NM_001371596.2), *AGTPBP1* (NM_001330701.2), and *APTX* (NM_001195248.2)” genes, and three variants have been previously reported in “*TPP1* (NM_000391.4), *AGTPBP1*(NM_001330701.2), and *PCDHGC4* (NM_018928.3)” genes. These genes are associated with AR NCL type 2, AR NCL type 7, AR CONDCA, AOA1, and NEDGS, respectively [21]. Three patients in our cohort were diagnosed with NCL: patient 1 (NCL type 2) and patients 5 and 6—siblings (NCL type 7). To date, 131 variants have been reported in the *CLN2* gene which is distributed over the whole protein structure. These include missense variants (63, 48%) followed by frameshift (21, 16%) and nonsense (17, 13%) variants. Two known common pathogenic variants, c.509–1 G > C and c.622 C > T p.(Arg208*), occur in 60% of affected individuals with NCL2 [22]*.* WES analysis revealed a splice site variant in exon 9 of the *TPP1* gene in patient 1. Our patient presented at the age of 3 years with GDD which was followed by progressive visual impairment, motor disability, spasticity, and scoliosis. NCL type 2 typically presents with seizures and/or ataxia in the late-infantile period by the age of 2–4 years, often in combination with a history of speech delay, followed by progressive childhood dementia, motor and visual impairment, and early death [23]. Our findings agree with previous reports that studied the clinical characteristics of NCL2 patients [24–26]. However, our patient had progressive spastic quadriplegia and scoliosis so there was a clinical overlap with other conditions such as hereditary spastic paraplegia [27]*.*

A novel (c.638C > A; p.Pro213Gln) in the *MFSD8* gene in a homozygous state was detected in patients 5 and 6. Stogmann et al. (2009) reported an Egyptian family with late-infantile seizures, deterioration, and loss of psychomotor skills 1 year after the seizures’ onset. Additionally, they had aggressive behavior, memory impairment, and language abnormalities with substantial loss of speech function [28]. In our study, we have observed marked intra-familial disease variability as both patients (P5 and 6) had different age of onset. Also, one of the two probands (P6) presented with scoliosis. Several previously reported studies showed inter and intrafamilial phenotypic variability for the same genotype in different forms of NCLs which agrees with our study [29]. The clinical heterogeneity may be related to the profoundly different disease mechanisms, the presence of modifier genes, other environmental factors, or lifestyle. Modifier genes could influence gene expression levels and post-translational processing [30]. The current computational analysis showed that Pro213Gln is highly conserved with potential pathogenicity impact on the transportation function of *MFSD8* protein accumulating the diseased harmful compound.

Several NCL disease-specific therapies have been identified depending on the unique subtype identified. These therapies range from several options in the CLN2 subtype such as enzyme replacement therapies, gene therapies, stem cell therapies, and pharmacological drugs to no available options in the CLN7 subtype rendering the identification of each type of particular importance [31].

Patient 2 was diagnosed with NEDGS due to a pathogenic homozygous missense variant in the *PCDHGC4* gene (c.1463C > T; p.Ala488Val). This syndrome was first described in a cohort of 19 patients from nine unrelated families originating from Iran, Iraq, Turkey, Morocco, Pakistan, Saudi Arabia, Lebanon, Sudan, and Jordan. It is characterized by the presence of dysmorphic features, neurodevelopmental delay, microcephaly, short stature, seizures, hypotonia, spasticity, strabismus, and skeletal anomalies [32]. Our patient had a similar presentation apart from cardiac anomalies (ASD) that was not previously reported. Five nonsense, frameshift, or splice site mutations were predicted to result in premature termination and a loss of function, and three missense mutations at highly conserved residues were reported. To our knowledge, this is the 2nd study that has detected a pathogenic variant in the *PCDHGC4* gene. Both patients 3 and 7 had novel homozygous mutations in *AGTPBP1*. A gene that was first described by Shashi et al. [33] in 13 individuals from 10 unrelated families with abnormal eye movements, GDD, microcephaly, tongue fasciculation, hypotonia, muscle atrophy, feeding difficulties, and failure to thrive. All patients had cerebellar atrophy; hence, it was subsequently recognized as CONDCA [34, 35]*.* Another study reported a similar phenotypic presentation but without any eye movement abnormalities [36]. Our computational studies showed a potential pathogenicity effect of the Gly884Arg variant concluding it might lead to altered protein conformation and inhibit deglutamylation of tubulin and non-tubulin target proteins.

To our knowledge, this is the third clinical study reporting patients with CONDCA. Our patients had similar presentation but patient (3) had additional unique phenotypic features “dysmorphic features, seizures and skeletal deformities” that were not previously reported. Patient (7) had areflexia in the lower extremities due to axonal motor neuropathy as confirmed by nerve conduction and electromyography studies possibly due to Purkinje cell degeneration and motor neuropathy that has been described in this disorder. Brain MRI in our patients showed cerebellar atrophy which is the hallmark of this disorder that should be carefully interpreted given their clinical presentation to avoid confusion with other disorders such as pontocerebellar diseases that are associated with cerebellar hypoplasia [33]. The current computational analysis showed that p.Gly884Arg might lead to altered protein conformation inhibiting deglutamylation of tubulin and non-tubulin target proteins.

*APTX* variant, identified in patient 4, was first described by Aicardi et al. [37] as the cause of a syndrome mimicking ataxia telangiectasia that was named AOA1. Anheim et al. found that AOA1 was the fourth most common cause of AR cerebellar ataxia [38]. The p.Pro206Leu variant was the most frequent variant described worldwide [39]. Oculomotor apraxia was not a constant finding in all reported patients. Our result was in agreement with the previous studies regarding most of the clinical manifestations; however, our patient had congenital heart disease and poor retinal function that were not previously reported in *APTX* gene mutations thus expanding its phenotypic spectrum.

To our knowledge, this is the first genetic study of its kind from North Africa “Egypt” exploring the possible molecular defects underlying the overlapping NDs phenotypes. We identified three novel pathogenic mutations and expanded the phenotypic spectrum with newly associated clinical phenotypic findings in the studied patients.

In conclusion, this study highlights the importance of genetic testing for patients presenting with ND disorders where phenotypic characterization might not be sufficient for proper classification and early disease identification, especially for potentially treatable ones.

### Supplementary Information

Below is the link to the electronic supplementary material.

Supplementary file1 (JPG 43 KB) Supplementary Fig. 1 Functional Enrichment analysis representing (A) GeneMANIA results showed the significant functional relationship among the affected gene with false detective rate (FDR) scores, below each gene listed the interacted partner genes physically. The genes with dark green shadow represent a set of genes that share the non-recombination repair process, while the genes with brown shadow represent a set of genes that share the double-strand break repair process. (B) PPI enrichment network among the affected genes showed that two human phenotypes were significantly enriched in 4 out of 5 analyzed proteins, the highlighted color corresponded with each phenotype.

Supplementary file2 (DOCX 20 KB)

## Data Availability

Availability of variant data during the current study has been submitted to the LOVD database under the following links; NM_001371596.2:c.638C > A (MFSD8; p.Pro213Gln; Novel variant) Data available at https://databases.lovd.nl/shared/individuals/00433029. NM_018928.3:c.1463C > T (PCDHGC4; p.Ala488Val; Reported variant; rs775104626) ClinVar; pathogenic, and citation = 0 Data available at https://databases.lovd.nl/shared/individuals/00433030 NM_001195248.2:c.635C > T (APTX; p.Ala212Val; Novel variant) Data available at https://databases.lovd.nl/shared/individuals/00433031. NM_001330701.2:c.2650G > C (AGTPBP1; p.Gly884Arg; Novel variant) REPORTED PREVIOUSLY AS NM_001330701.2:c.2650G > A; p.Gly884Arg) with ClinVar = NA, and citation = 0 Data available at https://databases.lovd.nl/shared/individuals/00433032. NM_000391.4: c.1145 + 2 T > G (TPP1; c.1145 + 2 T > G; Reported variant; COSV100196937); splice_donor_variant (Int. 9) Data available at https://databases.lovd.nl/shared/individuals/00433033. NM_001330701.2: c.1534A > G (AGTPBP1; p.Thr512Ala; reported variant rs1375829417) ClinVar; NA, and citation = 0 Data available at https://databases.lovd.nl/shared/individuals/00433034
