# Supplementary figures and images for: The Diagnostic Value of Whole-Exome Sequencing in a Spectrum of Rare Neurological Disorders Associated with Cerebellar Atrophy
Source: Mol Neurobiol. 2023 Dec 28;61(8):4949–61. doi: 10.1007/s12035-023-03866-y (PMC11249754; doi:10.1007/s12035-023-03866-y)

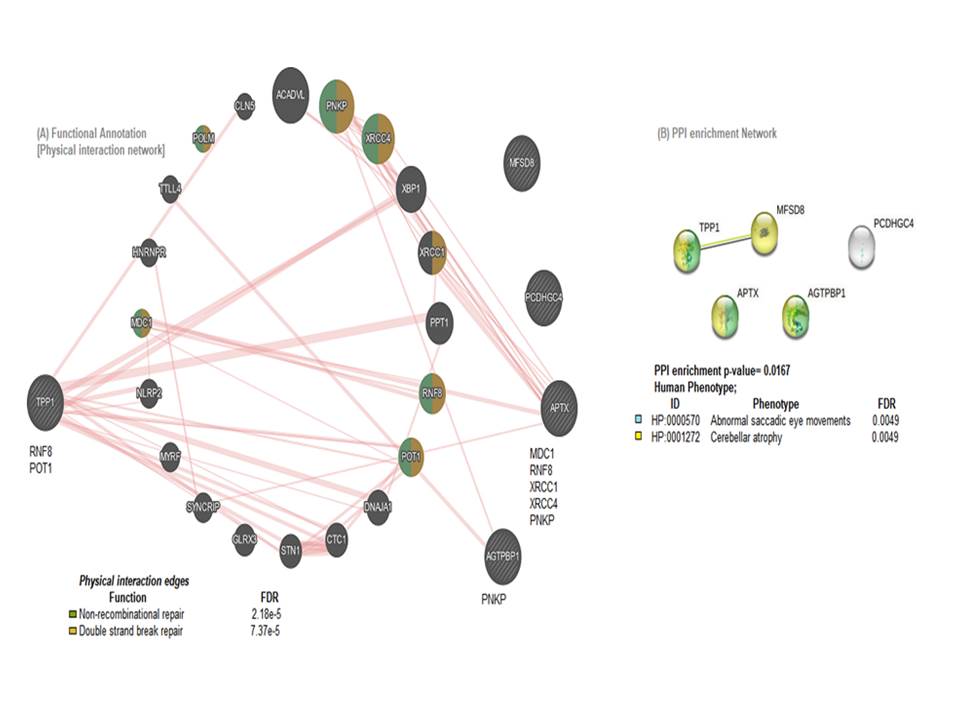

Supplement: Supplementary file 1 — Supplementary file1 (JPG 43 KB) Supplementary Fig. 1 Functional Enrichment analysis representing (A) GeneMANIA results showed the significant functional relationship among the affected gene with false detective rate (FDR) scores, below each gene listed the interacted partner genes physically. The genes with dark green shadow represent a set of genes that share the non-recombination repair process, while the genes with brown shadow represent a set of genes that share the double-strand break repair process. (B) PPI enrichment network among the affected genes showed that two human phenotypes were significantly enriched in 4 out of 5 analyzed proteins, the highlighted color corresponded with each phenotype. [file 12035_2023_3866_MOESM1_ESM.jpg]
