# Supplementary material for: The Diagnostic Value of Whole-Exome Sequencing in a Spectrum of Rare Neurological Disorders Associated with Cerebellar Atrophy
Source: Mol Neurobiol. 2023 Dec 28;61(8):4949–61. doi: 10.1007/s12035-023-03866-y (PMC11249754; doi:10.1007/s12035-023-03866-y)
Supplement: Supplementary file 2 — Supplementary file2 (DOCX 20 KB) [file 12035_2023_3866_MOESM2_ESM.docx]

**Supplementary Table 1: Multi-scale computational analysis results**

| **Tool** | **Score/Prediction** | **Implications** |
| --- | --- | --- |
| **NC_000004.12(NM_001371596.2):c.638C>A* (*MFSD8* gene; p.Pro213Gln; Novel variant)** | | |
| CADD / phred-like score | 24.7 / Likely harmful | Pro213Gln (P213Q) variant is highly conserved and it falls into the active transporter motif (PF00083), whose sensitivity to deleterious variants is reasonably high. Protein structure and clinical relevance-based prediction scores showed that Pro213Gln led to significant alteration in the protein function with disease-relevant impact.  **Finally, the predicted mechanism of action is that Pro213Gln might lead to transporter dysfunction for accumulating harmful compounds.** |
| SIFT | 0.03 / Deleterious |  |
| PolyPhen-2 | HumDiv= 0.994 / confidently damaging  Sensitivity (TPR)= 0.69; specificity (TNR): 0.97  HumVar=0.822 / confidently damaging  Sensitivity (TPR)= 0.74; specificity (TNR): 0.88 |  |
| BLOSUM62 | -1 / Conserved) |  |
| phastCons30way mammalian including human | 0.992 / Highly conserved |  |
| ClinPred (score) | 0.982 / Deleterious |  |
| DEOGEN2 (score) | 0.699 / / Deleterious  With PFAM log-odd score (PF) = 26.7  LOR (Log-Odd Ratio) = 22%. |  |
| MutPred score; based on Q8NHS3-1_human. | 0.841 / Altered Transmembrane protein (pvalue = 2.1e-04) |  |
| PROVEAN score | -3.12 / Probable damaging |  |
| VEST4 score | 0.937 / damaging effect |  |
| MUpro** | ∆∆G = -0.626 / DECREASE stability with high confidence) |  |
| BayesDel | (0.23 / Supporting Deleterious |  |
| GenoCanyon | 1 / Deleterious |  |
| fitCons | 0.7 / Deleterious |  |
| UniProt mining | Subcellular location; Helical transmembrane (212-232) |  |
| MotifFinder (In Pfam database) | MFS_1 (42-359)  **PF07690, Major Facilitator Superfamily;**  E-value = 1.1e-06  Sugar_tr (71-229)  **PF00083, Sugar (and other) transporter**  E-value = 9.5e-11 |  |
| **NC_000005.10(NM_018928.3):c.1463C>T (*PCDHGC4*; p.Ala488Val; Reported variant;** [**rs775104626**](https://www.ensembl.org/Homo_sapiens/Variation/Explore?db=core;tl=1nAaT0xZVvHJ0v8A-8595610;v=rs775104626)**) ClinVar; pathogenic, and citation = 0** | | |
| CADD / phred-like score | 24.4 / Likely harmful | Ala488Val (A488V) variant is highly conserved and it falls into the active domain called Codherin domain (PRU00043) that is responsible for cell-cell adhesion. Therefore, sensitivity to deleterious variants is reasonably high. Clinical relevance-based prediction score showed that Ala488Val has disease-relevant impact.  **Finally, the predicted mechanism of action is that Ala488Val might contribute in cell adhesion dysfunction.** |
| SIFT | 0 / deleterious |  |
| PolyPhen-2 | HumDiv=0.99 / confidently damaging  sensitivity (TPR)= 0.14; specificity (TNR)= 0.99  HumVar= 0.97 / confidently damaging  sensitivity (TPR)= 0.60; specificity (TNR)= 0.93 |  |
| phastCons30way mammalian including human | 0.974 / Highly conserved |  |
| ClinPred (score) | 0.974 / Deleterious |  |
| PROVEAN score | -3.55 / Damaging effect |  |
| MUpro** | ∆∆G = -0.4 / DECREASE stability with moderate confidence |  |
| UniProt mining | Located in Codherin 5 domain PRU00043 |  |
| **NC_000009.12(NM_001195248.2):c.635G>T (*APTX*; p.Ser212Ile; Novel variant)** | | |
| CADD / phred-like score | 23.5 / Likely harmful | Ser212Ile (S212I) variant is highly conserved and it falls into the active Histidine triad (HIT) domain, whose sensitivity to deleterious variants is reasonably high. HIT domain serve as catabolic enzymes acting on nucleotide‐containing substrates, where it is part of binding loop for the alpha-phosphate of purine mononucleotide. Hydrophobicity and solvent accessibility are most chemical properties contributors in Ser212Ile pathogenicity.  **Finally, the predicted mechanism of action is that Ser212Ile might lead to binding dysfunction with alpha-phosphate containing purine mononucleotide.** |
| SIFT | 0 / Deleterious |  |
| PolyPhen-2 | HumDiv= 0.880 / Confidently damaging  Sensitivity (TPR)= 0.68; specificity (TNR): 0.95  HumVar=0.878 / Confidently damaging  Sensitivity (TPR)= 0.73; specificity (TNR): 0.87 |  |
| phastCons30way mammalian including human | 0.986 / Highly conserved |  |
| BLOSUM62 | -2 / Conserved |  |
| FATHMM (score) | -4.04 / Deleterious |  |
| ClinPred (score) | 0.982 / Deleterious |  |
| DEOGEN2 (score) | 0.91 / Deleterious representing  PFAM log-odd score (PF) = 27.4  LOR (Log-Odd Ratio) = 19.7% |  |
| Hydrogen bonding capacity | (For Ser); with Lys214 and Ala215  (For Ile); with Ala215 |  |
| PROVEAN score | -4.89 / Likely damaging effect |  |
| VEST4 score | 0.616 / Likely damaging effect |  |
| Revel | 0.83 / Moderate deleterious |  |
| Varity | 0.94 / Deleterious |  |
| UniProt mining | HIT (Histidine triad ( domain (182-287)  PROSITE; PS51084; HIT_2 |  |
| BayesDel  Deleterious | 0.25 / Supporting pathogenic |  |
| fitCons | 0.71 / Deleterious |  |
| MetaLR | 0.9313 / Deleterious |  |
| **NC_000009.12(NM_001330701.2):c.2650G>C (*AGTPBP1*; p.Gly884Arg; Novel variant)**  **REPORTED PREVIOUSLY AS (NM_001330701.2:c.2650G>A; p.Gly884Arg) with ClinVar= NA, and citation=0** | | |
| CADD / phred-like score | 23.5 / Likely harmful | Gly884Arg (G884R) variant is highly conserved and it falls into crucial loop region for protein structure stabilization. Therefore, sensitivity to deleterious variants is reasonably high. Protein structure and clinical relevance-based prediction scores showed that Gly884Arg led to significant alteration in the bonding capacity with disease-relevant impact.  **Finally, the predicted mechanism** **of action is that Gly884Arg might lead to altered protein conformation inhibiting deglutamylation of tubulin and non-tubulin target proteins.** |
| SIFT | 0 / Deleterious |  |
| PolyPhen-2 | HumDiv= 1 / confidently damaging  Sensitivity (TPR)= 0.80; specificity (TNR): 0.98  HumVar=0.998 / confidently damaging  Sensitivity (TPR)= 0.81; specificity (TNR): 0.95 |  |
| phastCons30way mammalian including human | 0.999 / Highly conserved |  |
| BLOSUM62 | -2 / Conserved) |  |
| ClinPred (score) | 0.998 / Deleterious |  |
| MutPred score; based on Q9UPW5-1 human | 0.787 / supporting pathogenic  Loss of Loop (P = 0.05)  Altered Transmembrane protein (P = 8.3e-03) |  |
| Hydrogen bonding capacity | (For Arg); with Lys638  (For Ile); with Lys638 |  |
| Hydrophobic bonding capacity | (For Arg); with Lys638  (For Ile); Null |  |
| PROVEAN score | -6.75 / likely damaging |  |
| VEST4 score | 0.862 / Damaging |  |
| Varity | 0.84 / Deleterious |  |
| GenoCanyon | 1 / Deleterious |  |
| fitCons | 0.71 / Deleterious |  |
| **NC_000011.10(NM_000391.4: c.1145+2T>G** **(*TPP1*; c.1145+2T>G; Reported variant;** [**COSV100196937**](https://www.ensembl.org/Homo_sapiens/Variation/Explore?db=core;tl=1nAaT0xZVvHJ0v8A-8595610;v=COSV100196937)**) ; splice_donor_variant (Int. 9)** | | |
| MaxEntScan | Ref= 10.858  Alt=**3.211**  Diff=7.647 | **This variant has reasonable potential effect to diminish the splicing donor site to be skipped, thus alternative non-functional isoforms might be produced** |
| BayesDel | 0.66 / Deleterious |  |
| SpliceAI | Splice-Altering = 0.99 |  |
| dbscSNV Ada | 1 / Deleterious |  |
| dbscSNV RF | 0.92 / Deleterious |  |
| GenoCanyon | 1 / Deleterious |  |
| **NC_000009.12(NM_001330701.2):c.1378A>G ( *AGTPBP1*; p.Thr460Ala; reported variant rs1375829417) ClinVar; NA, and citation= 0** | | |
| CADD / phred-like score | 24.2 / Likely harmful | Thr460Ala (T460A) variant is highly conserved with decreasing impact on the protein stability. However, it isn't falls into known motifs or domains. Therefore, sensitivity to deleterious variants is reasonably accepted.  **Finally,** **the predicted mechanism** **of action is that Thr460Ala might lead to altered the protein stability. Hence, the degradable effect is predicted.** |
| SIFT | 0.03 / Deleterious |  |
| PolyPhen-2 | HumDiv= 0.842 / confidently damaging  Sensitivity (TPR)= 0.83; specificity (TNR): 0.93 |  |
| phastCons30way mammalian including human | 0.962 / Highly conserved |  |
| MutPred score; based on Q9UPW5-1 human | 0.372 / Conserved |  |
| GenoCanyon | 1 / Deleterious |  |
| fitCons | 0.73 / Deleterious |  |
| MUpro** | ∆∆G = -0.57 / DECREASE stability with high confidence |  |

* All the described variants were annotated using chromosomal descriptions and were checked by LUMC mutalyzer v. 3.0.4 according to GRCH38 human genome assembly.

** Prediction score using SVM and sequence information
